# Méningite à *Haemophilus influenzae* de type non b chez un nourrisson: cause rare à pronostic réservé

**DOI:** 10.11604/pamj.2018.30.164.15371

**Published:** 2018-06-22

**Authors:** Meriem Rachidi, Fatima Azzahraa Moussair, Naima Daoudi, Nabila Soraa

**Affiliations:** 1 Service de Microbiologie, CHU Mohammed VI, Faculté de Médecine et de Pharmacie, Université Cadi Ayyad, Marrakech, Maroc

**Keywords:** Haemophilus influenzae de type non b, méningite, nourisson, Non-type b Haemophilus influenzae, meningitis, infant

## Abstract

Non-type b *Haemophilus influenzae* strains are a rare cause of invasive secondary involvement in infants. We here report the case of a 11-month old infant with meningitis caused by non-type b *Haemophilus influenzae*, a polymorphic gram-negative bacillus exceptionally described in the literature, of unknown origin and with fatal outcome. Clinicians and microbiologists should suspect these uncommon serotypes as a cause of meningitis.

## Abstract (en)

Non-Type b Haemophilus is a rare cause of invasive secondary localization in young children. We here report the case of a child aged 11 months old who had Meningitis due to Non-Type b Haemophilus, a gram -negative bacilli of polymorphous appearance still exceptionally described in the literature, whose origin was undetermined and whose evolution was fatal. Clinicians and microbiologists should suspect the presence of these infrequent serotypes, especially on a particular case.

## Introduction

Les méningites bactériennes de l'enfant constituent un problème de santé publique dans de nombreux pays, notamment en Afrique, de par leur fréquence et leur létalité. L'*Haemophilus influenzae* demeure l'un des principaux agents bactériens responsables d'infections qui peuvent etre invasives et graves, principalement chez les enfants de moins de 5 ans [1].La forme capsulée de sérotype b est la plus fréquente, les autres sérotypes étant exceptionnels au cours des méningites. La forme non capsulée est retrouvée dans les manifestations localisées de l'adulte et de l'enfant plus particulièrement oto-rhino-laryngologiques (ORL) et bronchopulmonaires [2]. Nous rapportons le cas d'une méningite à *Haemophilus influenzae* non b présentant une sensibilité aux C3G chez un nourisson admis aux urgences et hospitalisé en Réanimation Pédiatrique pour un tableau clinique évoquant un syndrome méningé fébrile compliqué de choc septique en soulignant la rareté de l'Haemophilus influenzae non b dans les méningites bactériennes du nourisson ,ainsi que l'apport de l'examen cytobactériologique du liquide céphalorachidien (LCR) pour redresser le diagnostic et orienter l'antibiothérapie.

## Patient et observation

**Figure 1:**
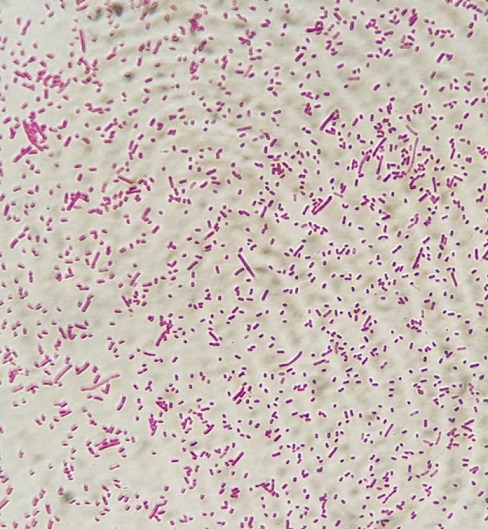
Examen direct: bacilles à gram négatif en diplocoque d’aspect polymorphe

**Figure 2:**
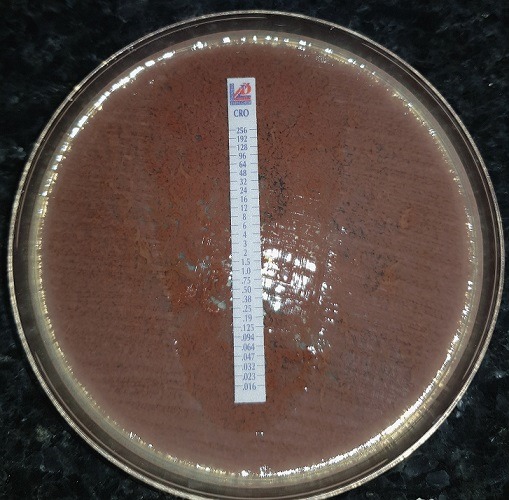
Concentration minimale inhibitrice Ceftriaxone

Il s'agit d'un nourisson de 11 mois qui aété pris en charge initialement au service de pédiatrie pour des convulsions généralisées fébriles d'installation brutale avec sepsis. Dans ses antécédents médicaux, la famille rapporte la vaccination selon le programme nationl établi. L'examen clinique général à l'admissiona objectivé un nourisson hypotone et somnolent tachycarde à 170 battements /min, polypnéique à 63 cycles / min, fébrile à 39.1°C, avec un syndrome méningé (hypotonie, signe de brudzinski et kerning positifs et des vomissements en jet) avec des marbrures au niveau des membres inférieurs. Une ponction lombaire réalisée a montré un LCR trouble avec des leucocytes à 38400 /mm^3^ dont 95% de polynucléaires neutrophiles. L'examen direct a retrouvé des bacilles à gram négatif en diplocoque d'aspect polymorphe (Figure 1). L'étude biochimique du LCR a montré une glucorrachie à 0.12g/l et une proteinorrachie à 7g/l. Le bilan infectieux était positif avec une CRP à 157mg/L et une hyperleucocytose à 8000/mm^3^. Le nourissona été réhydraté et mis sous une biantibiothérapie empirique à base de C3G (100mg/kg/j) et gentamycine 3mg/kg/j en attendant le résultat de la culture et l'antibiogramme avec surveillance stricte de son état neurologique. La patiente a été hospitalisée en Réanimation pédiatrique pour aggravation de son tableau clinique. L'examen clinique général a objectivé un nourisson inconsciente avec un SG = 12/15 hypotendu et eupnéique avec une fontanelle antérieure bombonte, un aspect en couché de soleil des yeux, des marbrures des extrémités et une hypotonie généralisée évoluant dans un contexte d'apyréxie à 36,5.La prise en charge a consisté en une mise en condition avec une intubation trachéale et sédation et une antibiothérapie à base de C3G par voie parentérale. La culture était positive à *Haemophilus influenzae* sensible aux pénicillines et aux C3G avec une CMI au céfotaxime sensible à 0,016 mg/l et une sensibilité aux antibiotiques suivants: bêtalactamines (amoxicilline, amoxicilline acide clavulanique, céfalotine, céfotaxime, imipenème)-Tétracycline-Pristinamycine et ciprofloxacine (Figure 2). Le complément par l'agglutination a révélé un Haemophilus influenzae de sérotype non b. L'ionogramme n'a révélé aucune anomalie. Le bilan infectieux de contrôle était positif avec une CRP à 79mg/L et une hyperleucocytose à 9000/mm^3^. L'identification bactérienne a reposé sur les caractères morphologiques, culturaux et biochimiques en utilisant les galeries API NH de Biomérieux ainsi qu'au test du satellitisme. L'étude de la sensibilité aux antibiotiques a été faite selon les normes du comité d'antibiogramme de la Société Française de Microbiologie (EUCAST) -CASFM). Une sérologie HIV réalisée était négative.Deux jours après son transfert en réanimation, l'évolution a été marquée par le décès du nourrisson suite à une défaillance hémodynamique et un arrêt cardiorespiratoire. L'exploration d'un éventuel déficit immunitaire associé n'ai pas été réalisé devant l'instabilité hémodynamique et le décès du patient.

## Discussion

Isolé en 1883 par Koch dans un pus de conjonctivite, puis en 1893 par Pfeiffer dans les expectorations de malades atteints de la grippe, *Haemophilus influenzae* (*H. influenzae*) a longtemps été considéré comme l'agent étiologique de la grippe. L'homme est le seul réservoir connu, avant la généralisation de la vaccination contre l'Hib, les enfants de moins de 5 ans constituaient le réservoir primaire du germe, avec un taux de colonisation du nasopharynx de 3 à 9% [3]. Depuis la vaccination, ce portage a considérablement diminué, actuellement, les enfants plus âgés et les adultes sont plus susceptibles d'abriter le germe (porteurs asymptomatiques) et forment probablement le réservoir primaire [2]. *Haemophilus influenzae* est un petit bacille à Gram négatif, immobile, non sporulé, polymorphe, souvent coccobacillaire, parfois filamenteux. Certaines souches peuvent présenter une capsule qui accroît leur virulence ou des fimbriae qui leur confèrent des propriétés d'adhésion et d'hémagglutination.Il est aéroanaérobie facultatif possédant une nitrate réductase et c'est un germe exigeant, ne poussant que sur des milieux de culture enrichis avec apport des deux facteurs indispensables à sa croissance : l'hémine (facteur X) et le NAD (nicotinamide adénine dinucléotide ou facteur V). La croissance de ces bactéries est favorisée par une atmosphère enrichie en CO2. Après 24 heures de culture à 37 °C, *Haemophilus influenzae* donne des colonies smooth, convexes, grisâtres, translucides de 0,5 à 1 mm. Les souches capsulées donnent des colonies mucoïdes de plus grosse taille [3]. Huit biotypes (I à VIII) ont été définis pour l'espèce *Haemophilus influenzae* [3, 4]. Le biotype I est plus fréquemment retrouvé dans les méningites et les manifestations invasives, le biotype II dans les infections bronchopulmonaires et les otites, le biotype IV dans les infections génitales [4].

La grande majorité des infections systémiques à *Haemophilus influenzae* est due aux souches capsulées de type b en raison du rôle majeur du polyribosephosphate (PRP) comme facteur de virulence (résistance à la phagocytose et à l'action du complément). Les autres sérotypes sont, en effet, éliminés au cours de la phase septicémique et présentent un risque moindre de localisations secondaires (arthrites, méningites). Un cas de méningite de type *Haemophilus influenzae* de sérotype a, publié au niveau de la revue Emerging Infectious Diseases en 2017, a été diagnostiqué chez un nourisson de 17 mois d'Oman en 2015 à l'hopital Nizwa, suivi pour déficit enzymatique en glucose-6-phosphate déshydrogénase (G6PD). Le patient a été traité par ceftriaxone intraveineuse. Le patient a eu une évolution clinique défavorable sous ceftriaxone qui a été remplacé par l'ampicilline par voie intraveineuse et son état s'est améliorée progressivement par la suite [5]. La méningite à *Haemophilus influenzae* de sérotype a est rapporté, surtout chez les enfants de 6 à 24 mois, autrement en bonne santé. Il a été signalé comme étant le plus virulent parmi les *H. influenzae* encapsulés après Hib [6, 7]. Une étude de surveillance hospitalière active sur la méningite de 1996 à 2007 à Salvador (Brésil)a rapporté que la méningite de Hia et de Hib survenait principalement chez les enfants de moins de 5 ans; les taux de létalité étaient supérieurs à ceux de la méningite causée par les souches e et f et NTHi, survenant dans les groupes plus âgés et ayant tendance à avoir un meilleur pronostic [5,8]. La méningite de Hia a été signalée principalement chez les peuples autochtones du Canada, de l'Alaska (USA) et de l'Australie et dans le sud-ouest des États-Unis; au Brésil, en Gambie, en Afrique de l'Est et en Papouasie-Nouvelle-Guinée. Des cas sporadiques ont été signalés dans le reste du monde. Les raisons des taux élevés de la maladie de Hia invasive chez les enfants autochtones restent floues. Au Canada, où la maladie invasive non *Haemophilus influenzae* a été incluse dans la liste des maladies à déclaration obligatoire depuis 2007, une initiative axée sur la santé publique a été établi pour mieux caractériser l'épidémiologie de la maladie invasive à Hia et développer un vaccin candidat contre Hia [5,9]. 3 cas d'infections à *Haemophilus influenzae* deux de sérotype a et une de type c ont été rapporté chez les adultes à Brooklyn, New York en 1980 [10]. Une étude faite au Portugal portant sur l'émergence de souches non encapsulées et encapsulées des isolats d'Haemophilus influenzae de sérotype non b entre 1989 et 2001 sur 119 isolats a concluque le taux de souches non encapsulées a augmenté (de 19 à 80%) avec la découverte de la première souche de *Haemophilus influenzae* sérotype f invasive [11].

## Conclusion

Cette observation rapporte le cas d'une méningite à *Haemophilus influenzae* non b chez un petit nourrisson ayant évolué vers le décès du patient malgré la sensibilité de la souche aux C3G et la prise en charge en réanimation. La réalisation rapide des prélèvements bactériologiques reste indispensable pour poser un diagnostic précoce et orienter l'antibiothérapie. Le sérotype non b reste exceptionnel en cas de méningite et présente un risque moindre de localisations secondaires ce qui souligne la nécessité de compléter par une identification du serotype de la souche isolée pour mieux caractériser ces souches souvent liées à un mauvais pronostic. La surveillance de l'incidence de la maladie invasiveà *Haemophilus influenzae* de sérotype non b s'avère ainsi indispensable.

## Conflits d’intérêts

Les auteurs ne déclarent aucun conflit d'intérêt.
